# Live Biomass of *Rigidoporus vinctus*: A Sustainable Method for Decoloration and Detoxification of Dyes in Water

**DOI:** 10.3390/microorganisms11061435

**Published:** 2023-05-29

**Authors:** Shehnaz, I. B. Prasher, Naushad Ahmad, Mukhtar Ahmed, Shivani Raghuwanshi, Vijay Kumar, Sharf Ilahi Siddiqui, Seungdae Oh

**Affiliations:** 1 Department of Environment Studies, Panjab University, Chandigarh 160014, India; chaudhryshehnaz92@gmail.com; 2 Department of Botany, Panjab University, Chandigarh 160014, India; chromista@yahoo.co.in (I.B.P.); shivaniraghuwanshi87@gmail.com (S.R.); vijaysharmasanmotra100@gmail.com (V.K.); 3 Department of Chemistry, College of Science, King Saud University, Riyadh 11451, Saudi Arabia; anaushad@ksu.edu.sa; 4 Department of Zoology, College of Science, King Saud University, Riyadh 11451, Saudi Arabia; mahmed1@ksu.edu.sa; 5 Department of Chemistry, Ramjas College, University of Delhi, Delhi 110007, India; 6 Department of Chemistry, Jamia Millia Islamia, New Delhi 110025, India; 7 Department of Civil Engineering, Kyung Hee University, Yongin-si 17104, Gyeonggi-do, Republic of Korea

**Keywords:** fungi, *Rigidoporus vinctus*, biosorption, methylene blue, Congo red

## Abstract

In this study, white-rot fungus, *Rigidoporus vinctus*, collected from an unidentified fallen twig from Pathankot, Punjab, India, was used for biosorption of anionic Congo red and cationic Methylene blue dyes from an aqueous medium. The biosorption efficiency of the live biomass of *Rigidoporus vinctus* was investigated to optimize biosorbent dosage, process time, concentrations of dyes, and pH of solutions. The results indicated that *Rigidoporus vinctus* is more efficient than other reported bio-adsorbents for Congo red and Methylene blue dyes. The maximum biosorption activity of *Rigidoporus vinctus* for Congo red was found at pH 2, and that for Methylene blue was at pH 10, after 24 h of the reaction period. The process followed pseudo-second-order kinetics, which indicated that the interaction of both dyes to the adsorption sites on the surface of *Rigidoporus vinctus* was responsive to biosorption. The biosorption process could be well explained by the Langmuir isotherm for both dyes. The maximum monolayer biosorption capacity of *Rigidoporus vinctus* for Congo red and Methylene blue was observed to be 54.0 mg/g and 80.6 mg/g, respectively. The seed germination test was carried out, and it was assessed that the toxicity of dyes was reduced up to significant levels. Based on the present experimental findings, it can be concluded that biosorption using the live biomass of *Rigidoporus vinctus* can effectively decolorize dye-containing wastewater, thus reducing the hazardous effects of dyes on human beings.

## 1. Introduction

The rapid growth of population, urbanization, and industrialization is creating serious environmental concerns, especially water pollution, and this problem is being faced worldwide [1]. The environment is under constant threat due to man-made activities that are polluting the water by releasing oily products, pharmaceuticals, toxins, heavy metals, coloring substances, and various other toxic chemicals [2].

Dyes are used widely in leather tanning, plastic processing, food processing, cosmetic preparation, rubber, and textile industries [3,4]. Commercially, more than 10 thousand types of dyes are available, with yearly production of more than 0.7 million tons [5]. The effluents discharged from the textile industries have been deemed to be a primary cause of groundwater pollution [6,7]. According to the World Bank reports, textile industrial effluents are responsible for 17–20% of total water pollution [8]. 

The accumulation of dyes in water bodies has severe implications, including a detrimental impact on water’s aesthetic qualities. Dye-containing water impacts aquatic creatures since dyes obscure light and disturb photosynthesis, as well as being dangerous to food chain organisms and even carcinogenic [9]. The dyes’ concentration in water, even at 1.0 mg/L, is responsive to the dark color of water, making the water unfit for domestic purposes [10].

Hence, dyes must be eradicated from wastewater discharged from the textile industries before it is released into natural water bodies [11]. To limit the hazardous effects of dyes on human beings and other living organisms, the legislations related to the environment are becoming stricter [12,13,14], and they even need to be amended.

There are numbers of physicochemical methods, such as coagulation/flocculation [14], oxidative degradation [15], filtration [16], ozonation [17], sonolysis [18], photocatalysis [19,20], and adsorption techniques [21], used to remove the dyes present in water. However, the primary disadvantages of physicochemical approaches are the huge cost, poor efficiency, limited versatility, intervention by other constituents of wastewater, and problems related to the handling of waste generated during treatment [22,23].

Nowadays, the use of nanomaterials as adsorbents is also gaining attention as they are more efficient, cost-effective, have high surface area, and are also environmentally friendly [24,25,26]. Many nanomaterials are used for adsorption of Methylene blue (MB), Congo red (CR), and other dyes [27,28,29,30,31,32].

Biological processes are gaining popularity over conventional chemical and physical methods for treating water containing dyes [22]. Therefore, there is a need to shift toward biological approaches as an eco-favorable option for the treatment of water containing dyes. The technique requires comprehensive investigation and advancement focused on using microbial organisms such as fungi [33], photosynthetic bacteria [34], and algae [35].

There are some advantages of using fungi for adsorption over other physicochemical methods, such as because of the extracellular, nonspecific, and nonselective enzyme system and cytochrome P450 [36]. The second advantage is the presence of the Fenton reaction, which naturally occurs in fungi and produces OH radicals that help in degrading the xenobiotics [37]. The third advantage is the presence of various functional groups such as hydroxyl, phosphate, and amine groups that provide the binding site for various molecules present in dyes [38].

In recent years, research on the decolorization of water using fungi has been reported with the huge ability to reduce the concentrations of a wide range of colors, and some of the fungi with their application in the adsorption of various dyes are listed in Table 1 and Table 2. The white-rot fungi, brown-rot fungi, and other fungi such as *Aspergillus niger*, *Rhizopus arrhizus*, and *Rhizopus oryzae* are examples of fungi that have shown an excellent ability to decolorize the colored water [39,40,41].

Decolorization of colored water using fungi involves mainly two mechanistic aspects, biosorption followed by enzymes of the live fungal biomass [60]. The production of enzymes is dependent solely on the nutrients, and dye decolorization capacity through biodegradation is based on its growth conditions. In addition to biodegradation, very little research on the decolorizing of wastewater through biosorption using fungi has been performed; therefore, more fungi with potential biosorption ability for synthetic dyes need to be explored. Due to the varied environmental variables involved in dye-containing wastewater, a high level of fungal screening is required for dye decolorization [61]. Because dye contains nitrogen oxides, sulfur oxides, and volatile organic components, it pollutes the air. The scraps of textiles, fabrics, and yarns, as well as discarded packaging, make up the majority of solid waste [62]. There are also some disadvantages of using fungi as adsorbents such as incomplete removal, generation of toxic sludge, and disposal of waste products [63].

This study has been performed to explore these fungal-based water treatment techniques, and for that live fungal biomass of *R. vinctus* was screened for the biosorption of two toxic dyes, cationic MB and anionic CR. There have been no earlier reports of *R. vinctus* being used to decolorize colored water.

Therefore, in the present study, *R. vinctus* was identified, and the physiochemical properties were monitored using spectroscopic and microscopic techniques. The live fungal biomass of *R. vinctus* was used in batch mode experiments for MB and CR biosorption. The effect of operating conditions, such as pH of dye solutions, fungus amounts, dyes concentration, reaction temperature, and contact time, has been investigated. The equilibrium data have been analyzed by fitting in various isotherms and kinetic relationships. The various parameters obtained from the investigation have been used to explain the mechanism, types of interaction, feasibility, and spontaneity of the process.

## 2. Materials and Method

### 2.1. Materials

**Figure 1:**
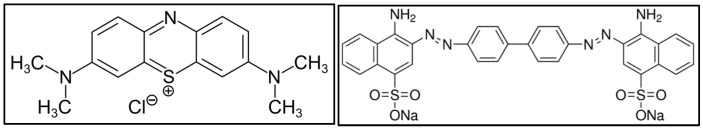
Structural image of MB (**left**) and CR (**right**).

The basidiocarp of *R. vinctus* was collected from an unidentified fallen twig from Pathankot, District of Punjab, India. MB (Figure 1; molecular weight = 320 g/mol, and λ_max_ = 665 nm) and CR (Figure 1; molecular weight = 696.664 g/mol, and λ_max_ = 500 nm) dyes were purchased from SISCO Pvt. Ltd., New Delhi, India. The nutrient media was obtained from Himedia Laboratories Pvt. Ltd., New Delhi, India. Acid, HCl, and base, NaOH, were purchased from Sigma-Aldrich, Mumbai, India. Experiments were performed with deionized water. A Nikon Eclipse E200 light microscope was used to observe microscopic structures of fungi. The Thermo Scientific-Evolution 201 UV/Vis-Spectrophotometer was employed to measure CR and MB concentrations at wavelengths 500 and 665 nm, respectively. The surface characterization of the fungus was carried out using a Field Emission Scanning Electron Microscope (FESEM) Hitachi SU8010 equipped with EDAX (Bruker 127 eV). Fourier-transform infrared spectroscopy (FT-IR), using a Perkin-Elmer (RXI) spectrophotometer, was employed to find the functional sites over the fungus surface and determine the interaction between fungal sites and dye molecules. Deluxe pH Meter-101 was used to determine pH by continuously measuring and adjusting the pH accordingly. The black chickpeas and green gram used for toxicity evaluation were purchased from a local market of sector 15 Chandigarh, India.

### 2.2. Methods

The pure culture of the fungus from the basidiocarp was obtained on malt extract agar (MEA) media (20 g malt extract, 20 g agar-agar, and 0.1 g of chloramphenicol in 1000 mL distilled water). The culture was maintained in MEA media before use and stored at 4 °C for further experimentation and was revived every four months. The 5–7-day-old live biomass from the fungal culture plate was used, and mycelial discs of weight 0.2 g each (cut from the margin of the culture plate) were inoculated aseptically in each flask. The 100 mg/L MB and CR stock solution was prepared separately and diluted to the required concentration for adsorption experiments. The pH value was adjusted by the addition of 0.1N HCl and 0.1N NaOH solutions. The experiments were carried out under sterile conditions in the dark at room temperature and natural pH. The effect of bioremediated and untreated dye solutions on the germination of black chickpeas and green gram was studied. The study material was sterilized using 0.1% HgCl_2_ solution for 5 min, washed 3–4 times in sterilized distilled water, and then soaked overnight in autoclaved distilled water. The study materials were soaked in the respective dye solutions (10 mg/L concentration) using the sterilized Petri plates, with distilled water as the control. The test was performed in triplicate. After eight days, germination and rootlet length were measured [64,65].

### 2.3. Identification of Isolate

The description of the morphology of isolated fungus was carried out by microscopic observations. The collected specimen was mounted in 4% KOH and CR (CR for staining) and checked by microscope. The DNA of pure fungal culture was extracted using the cetrimidetetradecyltrimethyl ammonium bromide (CTAB) method [66]. The Internal Transcribed Spacer (ITS) region of fungal ribosomal DNA is of great significance in distinguishing fungal species. The ITS-1 (5′-TCCGTAGGT-GAACCTGCG-3′) and ITS-4 (5′-TCCTCCGCTTATTGATATGC-3′) regions were amplified using PCR. Blast Multiple Alignment Tool (BLAST) algorithm was used to compare the obtained sequencing against the database present in NCBI (National Center for Biotechnology Information). The DNA sequence of the ITS region was submitted to GenBank (NCBI), and the phylogenetic tree was made using MEGA X software (version of 2021) [67].

### 2.4. Screening for Lignin-Modifying Enzymes (LME)

The clearance of Azure B was used to evaluate the ability of the selected strain to these enzymes [68,69]. The LME media was prepared by dissolving 0.5 g C_4_H_12_N_2_O_6_, 1.0 g KH_2_PO_4_, 0.5 g MgSO_4_.7H_2_O, 0.01 g yeast extract, 0.001 g CuSO_4_.5H_2_O, 0.01 g CaCl_2_.2H_2_O, 0.001 g Fe_2_(SO_4_)_3_, and 0.001 g MnSO_4_·H_2_O in 1 L distilled water. The media was mixed with 0.01 percent *w*/*v* Azure B and 2 percent *w*/*v* agar and autoclaved. One mL of a 20% *w*/*v* aqueous glucose solution, sterilized separately, was added to this medium. The media was transferred aseptically to Petri dishes and, after solidification, inoculated with the test fungus and incubated in the dark at 24 °C. For a total of 10 days, the plates were checked regularly.

### 2.5. Screening for Cellulolytic Enzymes

The basal media for determining cellulose-degrading enzyme activity was prepared by dissolving yeast extract (0.1 g), C_4_H_12_N_2_O_6_ (5 g), KH_2_PO_4_ (1 g), MgSO_4_.7H_2_O (0.5 g) and CaC1_2_.2H_2_O (0.001 g), carboxymethylcellulose (CMC) (1% *w*/*v* low viscosity), and 2% *w*/*v* agar in 1 L distilled water. The medium was sterilized by autoclaving and transferred into Petri dishes; the next day, the plates were inoculated with a disc of test fungus, and the Petri dishes were incubated at 24 °C. After 7 days, the Petri dishes were stained with a 2% CR solution for 15 min. The plates were de-stained with 1 M NaCl for 15 min. The clearance zone around the colony indicated positive results [70,71].

### 2.6. Determination of Adsorption Capacity and Removal Rate

To investigate the adsorption capacity of dyes in water, tests were conducted in a batch method using a set of 100 mL Erlenmeyer flasks containing 10 mL dye solutions at room temperature with different weights of biosorbent (0.2–1.0 g/L), biosorption periods (24–168 h), dye solution concentrations (10–50 mg/L), and pH (2–10). After performing each adsorption experiment, the biosorbent was separated from the dye solutions using centrifugation, and then the remaining concentration of dyes in the supernatants was determined by analyzing the absorbance, at absorbance maximum, λmax, 665 nm, for MB and 500 nm, for CR. Initial (*C_o_*) and final concentrations (*C_e_*) of the dyes were used in Equations (1) and (2) to estimate the percentage removal of dyes and maximum uptake capacity of biosorbent as follows [72]:

(1) $$R(\%)=\left(C_{o}-\frac{C_{e}}{C_{o}}\right)100$$

(2) $$Q_{e}=\frac{(C_{o}-C_{e})V}{m}$$

where *V* (L) is the volume of dye solution and *m* (g) is the amount of biosorbent. Isotherm and kinetic modeling were carried out using experimentally obtained MB and CR adsorption data for biosorbent *R. vinctus*.

## 3. Results and Discussion

### 3.1. Identification of Isolate

#### 3.1.1. Morphological Identification 

**Figure 2:**
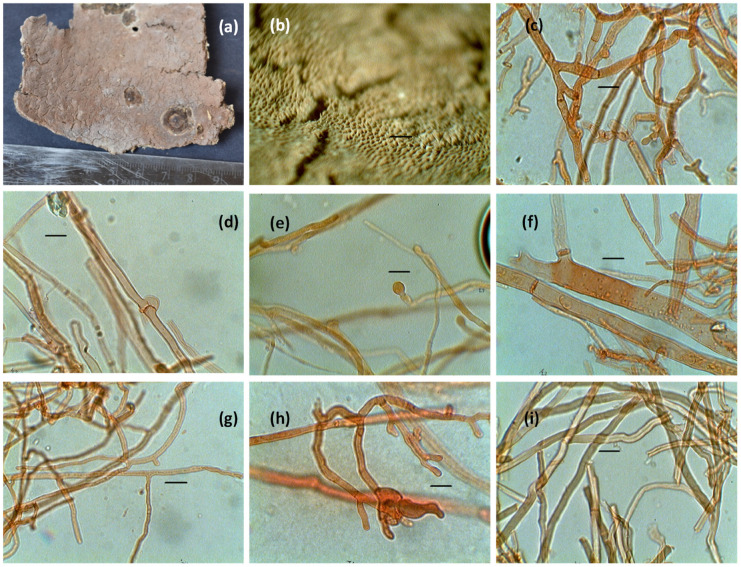
(**a**) Basidiocarp of *Rigidoporus vinctus*, (**b**) Pore surface, (**c**) Thin- and thick-walled, profusely branched, and septate hyphae, (**d**) Hyphae with clamp connection, (**e**) Hyphal pegs, (**f**) Hyphae with septa and oilglobules, (**g**) Thin-walled generative hyphae, (**h**) Branched hyphae, and (**i**) Skeletal hyphae.

A morphological identification was performed to identify the isolated fungal cells. Some of the morphological characteristics images of isolated fungi are shown in Figure 2a–i.

#### 3.1.2. Molecular Identification

**Figure 3:**
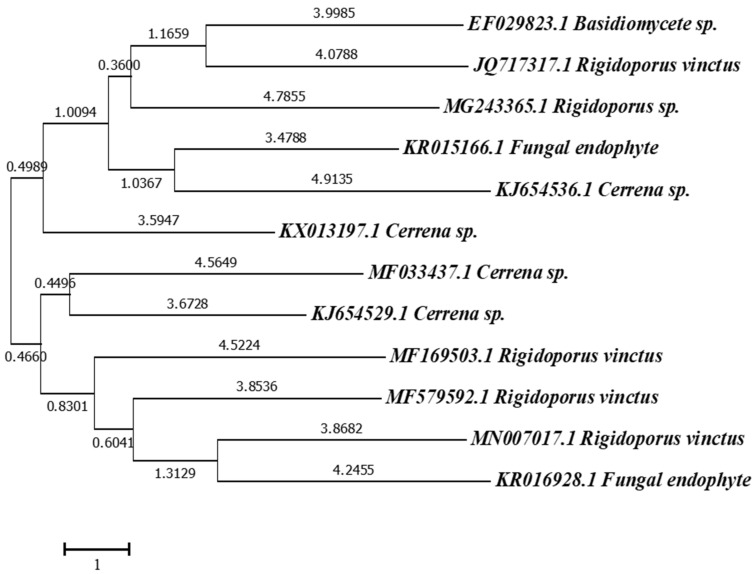
Phylogenetic analysis of partial ITS rDNA gene sequence of *Rigidoporus vinctus* and related microorganisms, (built with the help of MEGA 7.0 software by the neighbor-joining method with bootstrap values (1000 replicate runs)).

The molecular characterization showed 100% similarity to *R. vinctus*, and the phylogenetic tree was made on the basis of the sequence obtained after molecular characterization (Figure 3).

### 3.2. Screening for Cellulose-Degrading and Lignin-Modifying Enzyme Activity

**Figure 4:**
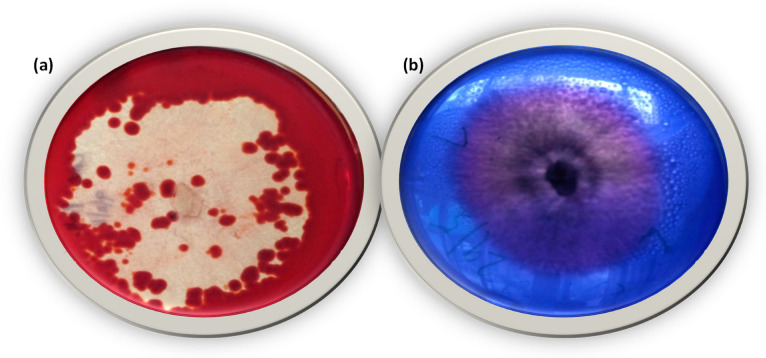
*Rigidoporus vinctus* (**a**) Cellulose-degrading enzyme activity, (**b**) Lignin-modifying enzyme activity.

The yellow opaque-colored zone emerged in the form of a concentric ring around the fungal growth, indicating cellulase activity (Figure 4a), in contrast to the red-colored un-degraded CMC. The decolorization of the blue color to an opaque purple color confirmed the production of ligninolytic enzymes (Figure 4b). Therefore, the isolated strain showed significant ligninolytic enzymatic activity and was thus further used for the removal of dyes.

### 3.3. SEM Analysis

**Figure 5:**
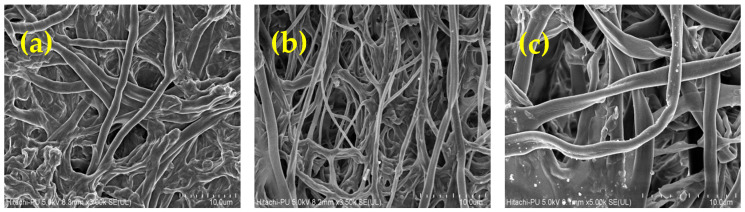
Images of SEM at 10 µm: (**a**) hyphae of fungi before adsorption, (**b**) hyphae of fungi after CR adsorption, and (**c**) hyphae of fungi after MB adsorption.

The surface morphology of *R. vinctus* (Figure 5a) showed that the fungus surface was quite irregular and formed by the agglomeration of hyphae. The surface showed roughness and cavities, which might have been favorable for the biosorption process. The cavities might have allowed the interaction of the MB and CR dyes with the surface of the *R. vinctus* [73]. From SEM images (Figure 5a–c), it is observed that before adsorption of dyes the fungal surface was clear and smooth, whereas after adsorption, dye molecules became attached on the hyphae surface and made the smooth surface of hyphae rough. A similar explanation is also given by other researchers [74].

### 3.4. EDS Study

**Figure 6:**
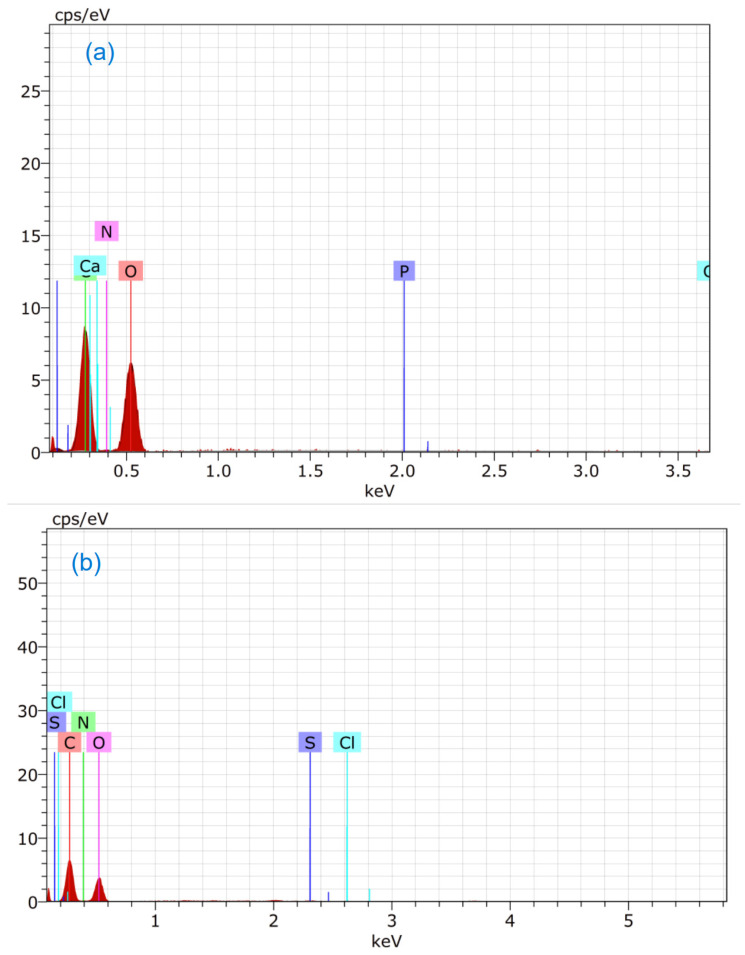

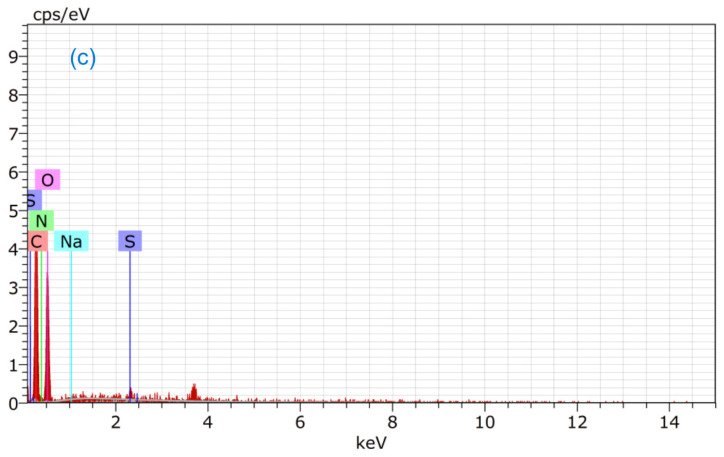
Spectra of EDS: (**a**) *R. vinctus*, (**b**) *R. vinctus* after MB adsorption, and (**c**) *R. vinctus* after CR adsorption.

The energy-dispersive spectroscopy (EDS) spectra before and after adsorption of MB and CR by fungi are shown in Figure 6a–c, and the numerical values are given in Table 3. The EDS investigation of *R. vinctus* before adsorption indicated the presence of the elements carbon, nitrogen, oxygen, calcium, and phosphorus in the fungus sample. EDS investigation after dye adsorption showed the presence of additional elements such as sulfur, chlorine, and sodium.

The fungus sample after MB adsorption showed the elements sulfur and chlorine, which indicated the presence of MB on the fungus surface. Similarly, the fungus that had CR adsorption showed the presence sodium and sulfur. In both the cases, the wt.% of carbon and nitrogen increased, showing dye molecules presence on the fungus surface.

### 3.5. FT-IR Analysis

**Figure 7:**
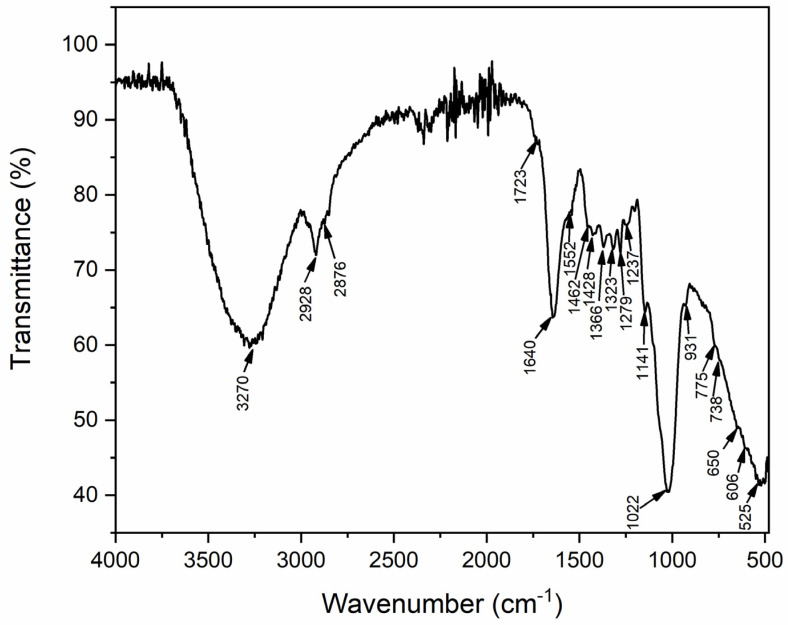
FTIR spectrum of *R. vinctus* fungal cells.

The FT-IR spectrum of *R. vinctus* (Figure 7) showed several absorption bands in the mid-IR region attributed to different functional groups present on the surface of *R. vinctus*. A broad peak at 3270 cm^−1^ was assigned to the –OH stretching vibration. The 2928 and 2876 cm^−1^ bands were attributed to the C–H stretching frequency. The shoulders at 2850–2500 cm^−1^ were assigned to the overtones and coupling between the in-plane bending O–H and C–O stretching vibrations of the fatty acids. The band at 1723 cm^−1^ appeared for the carboxyl C=O carboxyl stretching vibration. Several vibrational bands in the “fingerprint” region dominantly appeared for the protein structure or cell wall. The vibrational bands at 1640 and 1552 cm^−1^ were attributed to amide I (C=O stretching vibration) and amide II (N–H bending vibration), respectively. The band at 1462 cm^−1^ appeared for the asymmetric CH_3_ bending mode of the protein ethyl groups. The vibrational bands at 1430–1250 cm^−1^ were attributed to the amide III (C–N stretching and N–H plane bending). Vibration bands appeared at 775 cm^−1^, and 738 cm^−1^ might have been attributed to the amide IV (OCN bending distortion) and amide V (N–H out-of-plane bending), respectively. Amide VI (OCN bending) vibrational band was assigned at 650–525 cm^−1^. Various peaks from 1200–900 cm^−1^ were attributed to the polysaccharides in the cell structure [75]. These observations suggested that fungi contain several functional groups that may act as binding agents for charged molecules. Therefore, the present study attempted to quantify the interactions of organic dyes from water with *R. vinctus*. These results show good agreement with the previous literature [75].

### 3.6. Adsorption Studies

#### 3.6.1. Effect of *R. vinctus* Dosage and Contact Time

**Figure 8:**
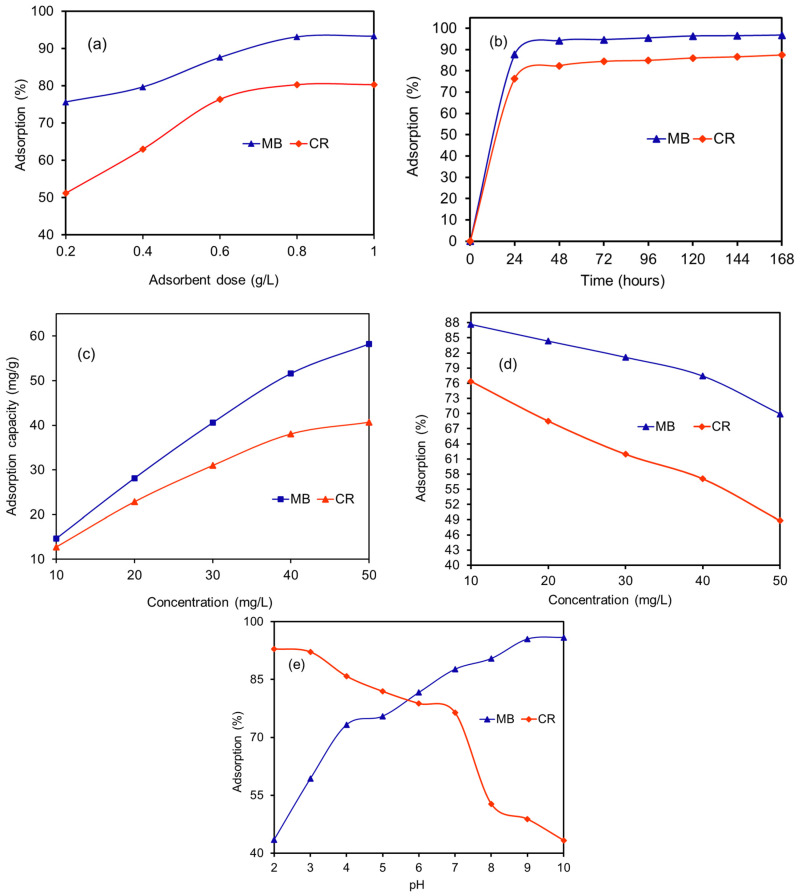
Effect of (**a**) adsorbent dosage, (**b**) contact time, (**c**,**d**) dye concentrations, and (**e**) pH on the adsorption of MB and CR.

The removal of CR and MB dyes through adsorption onto *R. vinctus* was studied using a dosage ranging from 0.2 to 1.0 g/L from the solution having an initial dye concentration of 10 mg/L at pH 7. The results (Figure 8a) showed that the removal of dyes had a direct correlation with the *R. vinctus* concentrations, and it could be estimated that the MB and CR removal efficiency of *R. vinctus* increased with an increase in the *R. vinctus* dosage. These observations were attributed to the increase in the availability of biosorptive sites with an increase in the *R. vinctus* dosage. From 51.5 to 76.4% of CR and from 75.7 to 87.6% of MB could be removed from the aqueous solution after increasing the *R. vinctus* dose from 0.2 g/L to 0.6 g/L; after this amount, no significant enhancement in the removal efficiency was observed. Therefore, this observed value was considered to be an optimized dose of *R. vinctus* for both MB and CR dye solutions having a 10 mg/L concentration. The obtained results also suggested a lower affinity of *R. victus* for anionic CR (10 mg/L) dye compared to cationic MB (10 mg/L) dye with the same concentration of *R. victus* at pH 7. This effect can be understood from the results of the effect of solution pH on CR and MB adsorption given in the pH section.

The contact time effect on the removal of CR and MB dyes was also studied to determine the design of the operating system (Figure 8b). The experiments were conducted with both MB and CR dye solutions with a 10 mg/L concentration, optimized amount of *R. vinctus* for different contact times (24–168 h). The rate of removal of color from the aqueous solution increased rapidly in the early stage (0 to 24 h). In the first 24 h, 87.6% MB and 76.4% CR were removed from the aqueous solution. After that, the removal rate dropped significantly, and after 48 h MB dye removal increased by only 6.5% to 94.1% removal, whereas CR removal increased by only 5.9% to 82.3% removal. After this, the dye removal rate became almost constant, and after 168 h a maximum of 96.7% of MB and 87.4% of CR dye could be removed. It is a well-known fact that, initially, all the sites on the *R. vinctus* surface might be vacant, which decreased and became fewer as time lapsed; thus, the coming molecules of dyes needed a specific pathway for adsorption, and these ultimately decreased the interaction of dyes as time passed. Similar behavior has been observed for the adsorption of dyes onto *Sargassum hemiphyllum* [76].

#### 3.6.2. Effect of Initial Dye Concentrations

These experiments were carried out with initial dye concentrations in the range of 10–50 mg/L, and a constant dose of 0.6 g/L. An increase in the concentrations of the dyes resulted in the higher collision of dye molecules to the adsorbent surface, therefore increasing the adsorbed amount of dye molecules to the biosorbent. For this study, the amount of adsorbed MB increased from 14.6 to 58.2 mg/g and for CR increased from 12.7 to 40.7 mg/g for 10 to 50 mg/L (Figure 8c). It has also been reported that a continuous increase in dye concentrations responded to a consistent decrease in the percentage of adsorption (Figure 8d). This effect might result from lowering in the adsorption sites for given higher concentrations; hence, a sufficient amount of dye molecules remained in the solutions as unadsorbed. These behaviors agree with previous works [9,77,78].

#### 3.6.3. Effect of Solution pH

The significant effects of solution pH on biosorption have been reported in the literature [79]. It has been reported that the change in the solution pH affects the charge of the biosorbent surface and the type of ionization of the dye molecules in water, which ultimately affects the biosorption of dye molecules [42]. The effect of the pH (2–10) of the dye solution was also investigated for the present biosorption study with a dye concentration of 10 mg/L and a *R. vinctus* dose of 0.6 g/L. As shown in Figure 8e, the percentage biosorption of CR increased toward the acidic pH. It achieved the maximum removal efficiency of 93% at pH 2.0, whereas the maximum percentage of biosorption of MB was 96% achieved at pH 10.0 (alkaline environment). The higher percentage of MB adsorption at the higher pH was because of the presence of low H^+^ ion concentration available for competing for sorption sites on the biosorbent, whereas at the low pH, the H^+^ ions on the *R. vinctus* surface largely made the surface positively charged, which decreased the biosorption of cationic MB ions [79]. In the case of anionic CR, the higher percentage of biosorption at the lower pH might be −CR to the positively charged *R. vinctus*. At the higher pH, the OH^−^ ions on the *R. vinctus* surface competed with the anionic CR, thus decreasing the biosorption percentage of CR dye in an alkaline environment [78]. These behaviors are in agreement with previously published works [42,80,81].

### 3.7. Isotherm Studies

To understand the techniques of sorption of MB and CR onto the fungal sites, the two important models, namely the Langmuir and Freundlich isotherms, were used to investigate the adsorption data obtained by varying the concentrations of dye solutions in the range of 10–50 mg/L.

**Figure 9:**
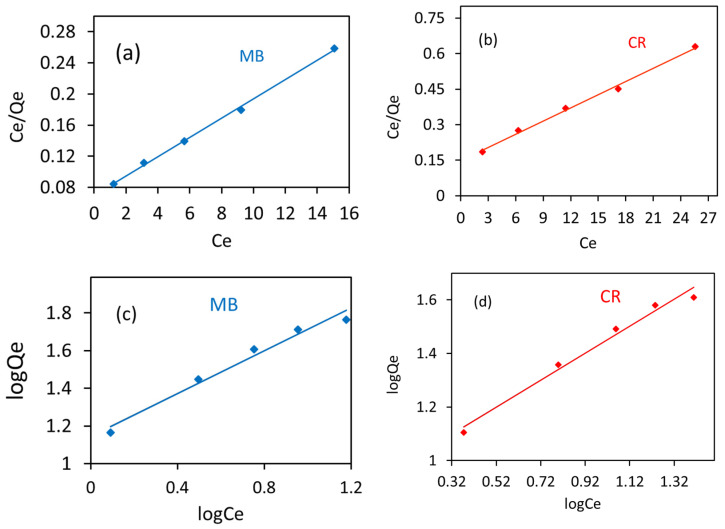

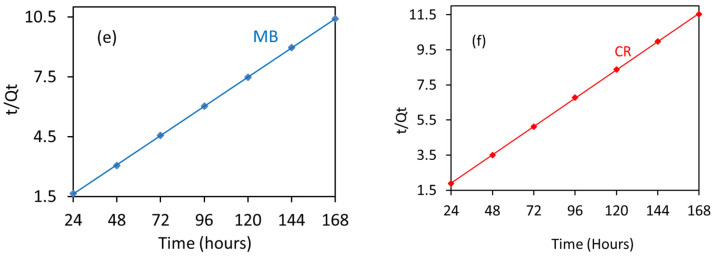
(**a**,**b**) Langmuir isotherm plots, (**c**,**d**) Freundlich isotherm, and (**e**,**f**) Pseudo-second-order plots.

The Langmuir model, used to explain the solid–liquid interface adsorption system, assumes the equivalent and independent adsorption sites on the *R. vinctus* surface, and no lateral interaction takes place during the adsorption process. The ultimate result of the Langmuir isotherm is homogeneous and monolayer adsorption of dyes on the *R. vinctus* surface. The Langmuir model can be defined as [82]:

(3) $$\frac{C_{e}}{Q_{e}}=\frac{C_{e}}{Q_{o}}+\frac{1}{Q_{o}b}$$

where *Q_o_* (mg/g) is the maximum monolayer adsorption capacity of a biosorbent, and *b* is the Langmuir constant related to adsorption energy. These Langmuir parameters can be calculated by plotting *C_e_*/*Q_e_* as a function of *C_e_*. For the present study, the Langmuir plots for MB and CR biosorption are shown in Figure 9a,b; the parameters were computed, and the numerical values are given in Table 4. The calculated values of the maximum adsorption capacity, *Q_o_*, for MB and CR were 80.6 and 54.0 mg/g, respectively. The adsorption capacity of *R. vinctus* was higher than reported in the literature (Table 2). The Langmuir constant, *b*, for MB had values of 0.177 L/mg, and that for CR was 0.124 L/mg. The regression coefficients, R^2^ (coefficient of determination), for MB and CR Langmuir plots approached the unity, 0.998 and 0.995, which indicated the good applicability of the Langmuir model for the explanation of adsorption phenomena. Unlike the Langmuir model, the Freundlich isotherm is used in that solid–liquid adsorption system where the solid surface is heterogeneous, and dye molecules form multilayers around the surface. The Freundlich model is defined as

(4) $$log\;Q_{e}=log\;kF+\frac{1}{n}\;log\;C_{e}$$

where *k_F_* (mg/g) (L/g)^1/n^ and *n* are Freundlich constants and area measure of adsorption capacity at unit concentration and adsorption intensity, respectively. The numerical value of *n* in the range 1–10 indicates the favorability of the sorption process. The plot of *logQ_e_* as a function of *logC_e_* gave values of Freundlich isotherm parameters. For the present study, the Freundlich plots for MB and CR are shown in Figure 9c,d, and the parameters calculated from these plots are given in Table 4. Freundlich constant (*n*) values were 1.76 and 1.77 for MB and CR, respectively, indicating the favorability of adsorption of both MB and CR onto the *R. vinctus* surface. The magnitude of *k_F_* can be taken as a relative measure of MB, and CR adsorption capacities onto the *R. vinctus* were found to be 13.9 and 16.7 (mg/g) (L/g)^1/n^ for MB and CR, respectively. The regression coefficients for the plots were 0.97 for both MB and CR. The regression coefficient of the Langmuir isotherm was closer to unity than the Freundlich model. Therefore, it can be concluded that the Langmuir isotherm was the best-fitted isotherm model for the sorption of both MB and CR dyes. The adsorption of MB on *Applantum lucidum* also followed the Langmuir isotherm model as reported by Naghipour et al. [42]. Nanthakumar et al. [83] also reported the better fitting of the Langmuir model than the Freundlich isotherm model for the adsorption of Reactive Blue 140 onto dead biomass of *Aspergillus niger*. The Langmuir adsorption isotherm was fitted well for the biosorption of Reactive dye by *Phanerochaete chrysosporium*, immobilized on a loofa sponge as reported by the literature [84]. Mustafa et al. [85] also reported the better fitting of the Langmuir model for the adsorption of reactive blue dye by *Panus tigrinus*.

### 3.8. Adsorption Kinetics

The determination of reaction kinetics is the most important tool to detect the actual mechanism behind the biosorption process, and it provides information for the appropriate water treatment system. Reaction kinetics involves various rate-controlling steps, which can be elaborated by fitting the time-dependent adsorption data to the various kinetics models [86]. The kinetics of the present process was examined by pseudo-first-order and pseudo-second-order models. These models can be defined as: 

Pseudo-first-order

(5) $$\log(Q_{e}-Q_{t})=\log Q_{e}-\frac{k_{1}}{2.303}t$$

Pseudo-second-order

(6) $$\frac{t}{Q_{t}}=\frac{1}{k_{2}Q_{e}^{2}}+\frac{t}{Q_{e}}$$

where *k*_1_ (1/h) and *k*_2_ (g/mg/h) are the rate constants, and *Q_e_* and *Q_t_* are the amounts of adsorbed molecules on the biosorbent sites (mg/g) at equilibrium and time *t*. The pseudo-first-order applies by assuming that only adsorption sites are responsible for the rate-determining step. In contrast, the pseudo-second-order model assumes that the adsorption rate depends on both adsorption sites as well as adsorbate molecules in the solution. The parameters of these models can be calculated by their linear plots, as shown in Figure 9e,f, which suggests the fitting of the appropriate model. For this study, the calculated parameters are given in Table 5. The obtained parameters are given the conformity of the best fitting of the linear plot of pseudo-second-order (Figure 9e,f) having an R^2^ value close to unity and showing the close values of calculated adsorption capacity (*Q_e_*, cal, for MB = 16.3 mg/g; and CR = 14.9 mg/g) to the experimental values (*Q_e_*, exp, for MB was approximately, 16.0 mg/g; and for CR it was approximately 14.0 mg/g). Similar behavior for biosorption of dyes was noticed in the previous literature [42,79,87,88]. Thus, from the fitting of pseudo-second-order, it can be concluded that the biosorption of MB and CR was a chemisorption process.

### 3.9. Proposed Biosorption Mechanism

**Figure 10:**
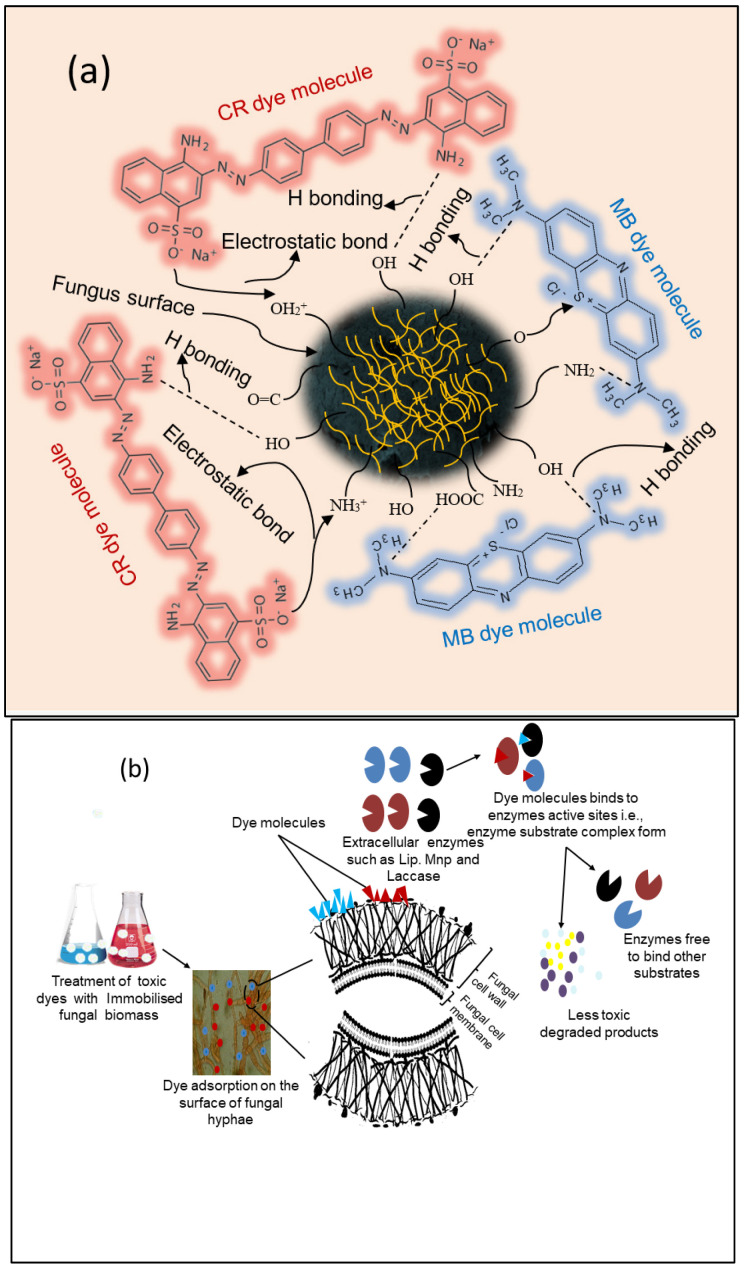
Proposed mechanism: (**a**) adsorption and (**b**) degradation of dye molecules onto the *R*. *vinctus* surface.

The proposed biosorption mechanism for the current study is shown in Figure 10. According to the previous literature [36,60], bioaccumulation, biosorption, and biodegradation are the three major steps in the dye removal process by fungi. Biosorption can occur in both live and dead biomass, whereas bioaccumulation occurs due to the metabolism of actively growing organisms. In general, electrostatic attraction, hydrogen bonding, and n-interactions are all potential proposed mechanisms for dye adsorption onto *R. vinctus* (Figure 10). The higher percentage of MB and CR sorption by the fungus might be due to different charged functional groups on the *R. vinctus* as shown by the FTIR spectrum of *R. vinctus* (Figure 7). The dye biodegradation begins when nonspecific extracellular and intracellular enzymes such as laccases, manganese peroxidases (MnP), and lignin peroxidases (LiP) are released from the fungi [36,60]. When dye molecules come into contact with fungal hyphae, they binds to the surface and induce the secretion of a variety of nonspecific extracellular and intracellular enzymes such as laccases, MnP, and LiP, which help in the degradation of the dyes and release the less toxic degraded products into the environment. Many studies have also shown that the cytochrome P450s (CYP) enzymatic system plays a role in the degradation of contaminants [36]. Some toxic molecules may be taken in by fungal cells and degraded by intracellular enzymes. In intracellular degradation, P450s play an important role in degradation. Fungi can use cytochrome P450 enzymes to metabolize a wide range of aliphatic, aromatic, and alicyclic chemicals, leading to hydroxylation, epoxidation, dealkylation, sulfoxidation, deamination, desulfuration, dehalogenation, and N-oxide reduction. The majority of P450s catalyze reactions after interacting with one or more protein components that transfer electrons [36,71,89].

### 3.10. Toxicity Assessment

**Figure 11:**
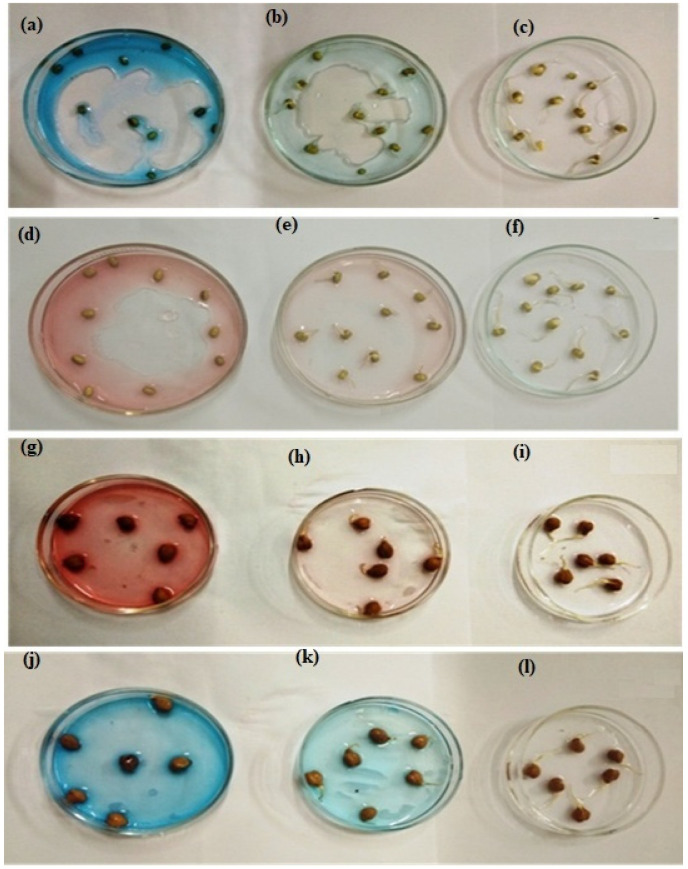
Germination of rootlets of seeds of *Vigna radiate* (**a**) in pure CR dye solution, (**b**) in treated CR dye solution, (**c**) in distilled water, (**d**) in pure MB dye solution, (**e**) in treated MB solution, and (**f**) in the distilled water. Germination of rootlets of seeds of *Cicer arietinum* (**g**) in pure CR dye solution, (**h**) in treated CR dye solution, (**i**) in distilled water, (**j**) in pure MB dye solution, (**k**) in treated MB solution, and (**l**) in the distilled water.

During the research, it was discovered that pure dye solutions, which are toxic, prevent seed germination, so rootlets do not emerge in *Cicer arietinum* and *Vigna radiate* seeds steeped in pure dye solutions. When *Cicer arietinum* and *Vigna radiate* seeds were steeped in a fungal-treated dye solution, tiny rootlets emerged from the seeds, indicating a decrease in toxicity. The average length of rootlets in the control was 3.5 and 3 cm, respectively, for *Cicer arietinum* and *Vigna radiate*, and when the study material was soaked in pure dye solutions (CR and MB), the rootlets were grown to a length of only 0.2 cm and 0.1 cm, respectively, for *Cicer arietinum* and *Vigna radiate*. When both dyes solutions were treated with fungi, the rootlets germinated to the length of around 2.5 cm and 2.3 cm, respectively, for black chickpeas and green gram (Figure 11) [65,71].

### 3.11. Comparative Studies

The isolated fungal cell revealed higher or comparative adsorption capacity to other adsorbents (Table 6) due to the high functionality and cellulosic structure of fungal cells. To help the young scientific community, herein, a number of recently used biosorbents have been cited.

## 4. Conclusions

The cultured fungi *Rigidoporus vinctus*, a white-rot fungus, has a number of functional groups on the surface; thus, it can adsorb organic dyes such as CR and MB from water. The obtained sorption data were fitted to various isotherms and kinetic models; the sorption data was well fitted to the Langmuir isotherm, and the values of maximum adsorption capacity, *Q_o_*, for CR and MB were found to be 54.0 mg/g and 80.6 mg/g, respectively. The reaction followed pseudo-second-order kinetics, which suggested the chemical interaction between the dyes and fungal sites. The comparative study showed that *Rigidoporus vinctus* has a higher sorption capacity than previously reported for other sorbents; therefore, the novel *Rigidoporus vinctus* represented excellent performance for colored water remediation. The results of the study suggested that due to the high dye removal efficiency of this novel biosorbent, *Rigidoporus vinctus* could further be used for the bioremediation of other recalcitrant compounds. A detailed study of the mechanism of bioremediation using live *Rigidoporus vinctus* is needed in the near future.

## Figures and Tables

**Table 1:** Biosorption of MB by fungi.

Order	Biosorbent	MB Adsorption Capacity (mg/g)	Reference
1.	Artist’s bracket fungi	100.0	[42]
2.	*Aspergillus carbonarius* *Penicillium glabrum*	21.88 16.67	[43]
3.	Edible fungus residue activated carbon	662.25	[44]
4.	*Aspergillus fumigatus* (dead biomass)	125.0	[45]
5.	*Rhizopusarrhizus* (dead biomass)	471.5	[46]
6.	Rice straw ferment with *Phanerochete chrysosporium*	51.4	[47]
7.	Dead biomass of *Fomesfomentarius* and *Phellinusigniarius*	204–232	[48]
8.	Dried biomass of *Aspergillus parasiticus*	63.29	[49]
9.	*Pleurotus ostreatus*-based biocomposite	40.11	[50]
10.	Spent mushroom waste	239.81	[51]
11.	Dried biomass of *Rhizopus arrhizus*	370.3	[52]

**Table 2:** Various fungi belonging to different groups used for adsorption of dyes along with their experimental conditions and biosorption %.

Order	Fungus	Dye	Removal Efficiency (%)	Experimental Conditions	Mechanism	Contact Time	Reference
**1.**	*Sphingomonaspaucimobilis*	Methyl red	99.63	pH 9.0 Temp. 30 °C Conc. 750 mg/L Shaking conditions	Enzyme production Biodegradation	10 h	[5]
**2.**	*Trametes versicolor*	Methyl red Reactive red 220	91.0 80.0	Conc. 75 mg/L Conc. 50 mg/L	Laccase and MnP	8 days	[33]
**3.**	*Applanatum lucidum*	MB	99.8	Initial conc. 25 mg/L pH 9.0 Biosorbent dose 0.15 g	Adsorption		[42]
**4.**	*Aspergillus fumigates*	MB	90.0	Initial conc. 12 mg/L pH 7–13 Temp. 20–22 °C		120 min	[53]
**5.**	*Pleurotus ostreatus*	MB	99.0	Initial conc. 70 mg/L pH 11 Biosorbent dose 0.1 g/L	Biosorption	24 h	[54]
**6.**	*Phanerochaete chrysosporium*	Orange II	85.0	Initial conc. 100 mg/L pH 5.0 Temp. 28–30 °C RPM 150	Ligninolytic enzymes	5 days	[55]
**7.**	*Trametes villosa*, *Trametes trogii* and *Coriolus versicolor*	Gentian violet Xylidine CR Malachite green Remazol brilliant blue R Indigo carmine Anthraquinone blue	13.0 23.0 40.0 46.0 82.0 94.0 95.0	Initial conc. 6.1 mg/L Initial conc. 12.8 mg/L Initial conc. 58.1 mg/L Initial conc. 7.0 mg/L Initial conc. 188.8 mg/L Initial conc. 23.4 mg/L Initial conc. 250 mg/L pH 4.5 Temp. 30 °C	Laccase and manganese peroxidase (MnP)	30 min	[56]
**8.**	*Lentinuscrinitus*	Reactive blue 220	95.0	pH 5.5 Temp. 28 °C Conc. 0.1 g/L	Biodegradation	10 days	[57]
**9.**	*Daldinia concentric* *Xylariapolymorpha*	Cibracron brilliant red 3B	-	Conc. 50 mg/L pH 4.5 Temp. 30 °C Static and shaking (150 rpm)	Laccase	5 days	[58]
**10.**	*Cerrena unicolor*	CR Methyl orange Remazol brilliant blue R Bromophenol blue Crystal violet	53.9 77.6 81.0 62.2 80.9	pH 4.5 Temp. 30 °C Conc. 100 mg/L	MnP	12 h 12 h 5 h 12 h 24 h	[59]

**Table 3:** Percentage composition of *R. vinctus* before and after adsorption of MB and CR.

Order	Element	wt.% of Fungus	wt.% of Fungus after Adsorption of MB	wt.% of Fungus after Adsorption of CR
1.	Oxygen	51.55	46.41	46.93
2.	Carbon	44.85	47.25	47.81
3.	Phosphorus	0.02	-	-
4.	Calcium	0.20	-	-
5.	Nitrogen	3.38	6.13	4.79
6.	Sulfur	-	0.12	0.42
7.	Chlorine	-	0.09	-
8.	Sodium	-	-	0.05

**Table 4:** Isothermal parameters for the adsorption of MB and CR at 27 °C.

Order	Pollutants	Langmuir				Freundlich		
		*Q_o_* (mg/g)	b (L/mg)	*R_L_*	R^2^	*k_F_* (mg/g)	N	R^2^
1.	MB	80.6	0.177	0.35	0.998	13.9	1.76	0.975
2.	CR	54.0	0.124	0.44	0.995	16.7	1.77	0.977

**Table 5:** Kinetics parameters for MB and CR adsorption at 27 °C.

Order	Parameters	Pseudo-First-Order			Pseudo-Second-Order		
		k_1_ 1/h	*Q_e_* (cal) mg/g	R^2^	k_2_	*Q*_e_ (cal) mg/g	R^2^
1.	MB	*Q_e_* (Exp.) = ~16.0 mg/g			*Q_e_* (Exp.) = ~16.0 mg/g		
		0.0002	2.6	0.948	0.0006	16.3	0.998
2.	CR	*Q_e_* (Exp.) = ~14.0 mg/g			*Q_e_* (Exp.) = ~14.0 mg/g		
		0.90	2.2	0.983	0.001	14.9	0.999

**Table 6:** Comparative study of MB and CR adsorption.

Order	Adsorbent	MB Adsorption Capacity (mg/g)	Reference
1.	Fe_2_O_3_-ZrO_2_/BC	38.10	[86]
2.	α-chitin nanoparticles	06.90	[90]
3.	Gum-arabic-coated Fe_3_O_4_	14.30	[91]
4.	Hydrogen titanate nanosheets	81.50	[92]
5.	Fe_3_O_4_@C nanoparticles	44.38	[93]
6.	Cu(OH)_2_-NP-AC	32.90	[94]
7.	CuO/MCM-41	65.70	[95]
8.	Acid-washed black cumin	73.53	[96]
9.	MnFe_2_O_4_/BC	10.07	[97]
10.	*Rigidoporus vinctus*	80.6	This study
		CR adsorption capacity (mg/g)	Reference
11.	Banana peel	1.72	[78]
12.	Bengal gram seed husk	41.66	[98]
13.	*Asprgillus niger*	14.16	[99]
14.	Cattail root	38.79	[100]
15.	Activated red mud	7.087	[101]
16.	Biowaste material	18.45	[102]
17.	Au-Fe_3_O_4_-NCs-AC	43.88	[103]
18.	Glycidyl methacrylate-g-poly (ethylene terephthalate fiber	16.60	[104]
19.	*Rigidoporus vinctus*	54.0	This study

## Data Availability

The datasets used and/or analysed during the current study are available from the corresponding author on reasonable request.
